# Microstructural and Texturizing Properties of Partially Pectin-Depleted Cell Wall Material: The Role of Botanical Origin and High-Pressure Homogenization

**DOI:** 10.3390/foods10112644

**Published:** 2021-11-01

**Authors:** Jelle Van Audenhove, Tom Bernaerts, Novita I. Putri, Erick O. Okello, Luisa Van Rooy, Ann M. Van Loey, Marc E. Hendrickx

**Affiliations:** Laboratory of Food Technology and Leuven Food Science and Nutrition Research Centre (LFoRCe), Department of Microbial and Molecular Systems (M2S), KU Leuven, Kasteelpark Arenberg 22, P.O. Box 2457, 3001 Leuven, Belgium; tom.bernaerts@kuleuven.be (T.B.); novitaika.putri@kuleuven.be (N.I.P.); omodhoerick@gmail.com (E.O.O.); luisavanrooy1@gmail.com (L.V.R.); ann.vanloey@kuleuven.be (A.M.V.L.); marceg.hendrickx@kuleuven.be (M.E.H.)

**Keywords:** fruits and vegetables, high-pressure homogenization, partial pectin depletion, cell wall material, rheology, water binding capacity

## Abstract

In the current study, the texturizing properties of partially pectin-depleted cell wall material (CWM) of apple, carrot, onion and pumpkin, and the potential of functionalization by high-pressure homogenization (HPH) were addressed. This partially pectin-depleted CWM was obtained as the unextractable fraction after acid pectin extraction (AcUF) on the alcohol-insoluble residue. Chemical analysis was performed to gain insight into the polysaccharide composition of the AcUF. The microstructural and functional properties of the AcUF in suspension were studied before HPH and after HPH at 20 and 80 MPa. Before HPH, even after the pectin extraction, the particles showed a cell-like morphology and occurred separately in the apple, onion and pumpkin AcUF and in a clustered manner in the carrot AcUF. The extent of disruption by the HPH treatments at 20 and 80 MPa was dependent on the botanical origin. Only for the onion and pumpkin AcUF, the water binding capacity was increased by HPH. Before HPH, the texturizing potential of the AcUFs was greatly varying between the different matrices. Whereas HPH improved the texturizing potential of the pumpkin AcUF, no effect and even a decrease was observed for the onion AcUF and the apple and carrot AcUF, respectively.

## 1. Introduction

A considerable amount of fruits and vegetables produced are processed into food products, such as drinks, soups, sauces and purees [1,2]. Edible plant tissue is generally built up by a hierarchy of structures as outlined by Waldron et al. [3], namely the cell, the cell wall, which is thin and non-lignified in the case of parenchymatous cells, and the polymers constituting the cell wall, being cellulose, hemicellulose, pectin and proteins. From a food technology standpoint, the cell wall properties have an important role in the quality perception of the textural properties of food products by consumers [3]. Traditionally, this cell wall is presented as a network of cellulose microfibrils cross-linked by hemicellulose surrounded by a matrix of pectin [4,5]. However, more recently, it has been shown that pectin can also interact with cellulose and hemicellulose [6,7,8]. Apart from the new insights on the interactions of pectin in the cell wall, Cosgrove [9] also argued that “biomechanical hotspots” exist, which are close interaction sites between cellulose microfibrils mediated by xyloglucan. Moreover, pectin, being the major constituent of the middle lamella, plays an important role in the adhesion between cells [10].

Generally, fruit- and vegetable-based dispersions are constituted of a dispersing phase (i.e., the serum phase) and a dispersed phase (i.e., the particle phase). Different factors could have an influence on the rheological properties of fruit- and vegetable-based food products, such as the composition of the serum phase (in particular, the pectin content, other soluble (cell wall) compounds, ions and the pH value), the particle concentration and the properties of the particles (in particular, their size distribution, morphology and deformability) [2,11]. Especially the concentration of particles in the dispersion has a great effect. At very low concentration, the particles flow through the serum phase without the potential of building a space-filling network between the particles, and thus, have a very small effect on the apparent viscosity. At this low particle concentration, the dispersion has a negligible storage modulus (G′). With increasing concentration, a network can be formed between the particles, resulting in a prominent increase in the apparent viscosity and the G′ of the dispersion [12,13]. In contrast to the relation between particle concentration and the G′ of the dispersion, which is rather straightforward, it is more difficult to relate other specific particle characteristics (e.g., size, morphology and deformability) to the resulting G′, due to the interrelations of these characteristics, and thus, the combined effect on the G′ [14]. In the region between the diluted and concentrated regime especially, an effect of distinct particle morphology (but also related with different sizes) on dispersion rheology was observed by Leverrier et al. [12], namely that cell clusters resulted in a higher apparent viscosity of apple puree than single cells because of the higher irregularity of the former, which improved the formation and rigidity of the network. A similar trend was observed for carrot dispersions, where the apparent viscosity decreased from cell clusters to single cells to cell fragments due to the differences in particle packing [15]. In this region between the diluted and concentrated regime, soluble polymers in the serum phase (e.g., the polymers natively present in the serum, carboxylmethyl cellulose or xanthan) had a significant contribution to the network formation. However, at high particle concentration, the G′ was almost completely determined by the stiff network formed by the particles, and the role of the polymers in the interstitial spaces was negligible [12,16].

The composition and microstructural properties of the serum and particle phase are dependent on the specific fruit or vegetable studied, which, in turn, has an effect on the resulting rheological and textural properties of the dispersion [1,17]. However, an even higher complexity arises when the effect of processing steps, for example thermal and mechanical treatments, on the rheological properties of fruit- and vegetable-based food products from different botanical origin are compared. Indeed, differences in the effect of these treatments on the microstructural and rheological properties have been shown before, which were attributed to differences in the composition and microstructural properties of the serum and particle phase between different fruits and vegetables [1,17,18,19,20]. High-pressure homogenization (HPH) represents a typical mechanical processing step, which could substantially alter the microstructural properties of these dispersions and, as a consequence, their rheological properties [21,22]. In the case of HPH, differences in composition and structure of the cell wall material (CWM) determine its resistance against the applied shear force in particular, and thus, the components that are potentially released [21]. For example, Lopez-Sanchez et al. [1] observed that HPH (one pass at 60 MPa) had an improving effect on the G′ of tomato dispersions, whereas a decrease in the G′ was found for broccoli and carrot dispersions with the same overall trend, independent of whether the dispersion was prepared by a cold or hot break process. In the case of broccoli and carrot, the decrease in the G′ was in line with the smaller particle size because of the breakdown of the cells. Although the particle size was also decreased in the case of tomato, the G′ increased by HPH for this matrix, which was attributed to a more prominent effect on the particle size and morphology because of substantial swelling of the fragments [1].

For lemon peel and pumpkin pomace, it is already shown that the texturizing potential of CWM could be improved by (partial) pectin depletion of the CWM and HPH [23,24]. After nitric acid pectin extraction (pH value of 1.6, 80 °C, 1 h) on the alcohol-insoluble residue (AIR) of lemon peel and pumpkin pomace, of which the conditions are in line with industrially relevant pectin extraction conditions [25], the G′ of the suspensions of the unextractable fractions were higher than of the suspensions prepared from the material without preceding pectin depletion and could be further improved by HPH [23,24]. In the current study, the texturizing potential of partially pectin-depleted CWM obtained as the unextractable fraction after nitric acid pectin extraction on the AIR, referred to as the acid-unextractable fraction (AcUF), was studied for an array of relevant fruits and vegetables. Furthermore, the potential of HPH to functionalize the pectin-depleted CWM of these matrices was investigated. Since the processing steps (including the pre-treatments) and analyses were performed in the same way, a straightforward comparison of the texturizing properties and the potential of functionalization by HPH between the apple, carrot, onion and pumpkin pectin-depleted CWM was facilitated. For this, aqueous suspensions of the partially pectin-depleted CWM of the different matrices were prepared. Of these model systems, the microstructure, water binding capacity (WBC) and viscoelastic properties in suspension were specifically addressed. However, the objective of the current study is beyond the comparison of this set of relevant matrices. Indeed, by including the partially pectin-depleted CWM of several fruits and vegetables with different chemical compositions and microstructural attributes, an important part of the discussion is focused on how the microstructure of partially pectin-depleted CWM is related to their texturizing potential. Furthermore, the effect of HPH at 20 and 80 MPa on these microstructural attributes and the potential to alter these texturizing properties are also addressed.

## 2. Materials and Methods

### 2.1. Materials

‘Jonagold’ apples (*Malus domestica*), yellow onions (*Allium cepa*), ‘Nerac’ carrots (*Daucus carota*) and ‘Hokkaido’ pumpkins (*Cucurbita maxima*) were bought at a local shop.

Technical ethanol (99%), technical acetone, Na_2_CO_3_ and NaOH pellets were bought from VWR (Leuven, Belgium). HCl, NaOH (0.1 M), H_2_SO_4_ (concentration ≥ 95% *w*/*w*) and disodium tetraborate decahydrate were obtained from Fisher Scientific (Merelbeke, Belgium). Rhamnose monohydrate, 3-phenylphenol and HNO_3_ were bought from Acros Organics (Geel, Belgium). H_2_SO_4_ (72% *w*/*w*) and NaOH (50% *w*/*w*) were obtained from Alfa Aesar (Kandel, Germany) and J.T. Baker (Gliwice, Poland), respectively. Galacturonic acid monohydrate and fucose were bought from Sigma-Aldrich (Diegem, Belgium), arabinose from Fluka Biochemika (Buchs, Switzerland), galactose from Merck (Darmstadt, Germany), glucose monohydrate from Riedel-de-Haën (Seelze, Germany), xylose from UCB (Leuven, Belgium) and mannose from Fluka Analytical (Buchs, Switzerland). The ultrapure water (organic free, 18.2 MΩ·cm resistance) was provided by a SimplicityTM 150 system. Unless otherwise mentioned, all chemicals used were of analytical grade.

### 2.2. Blanching of the Fruit and Vegetable Tissues

Before blanching, some preparative steps were performed on the apples, carrots, onions and pumpkins. The peels of the apples, carrots and pumpkins and the tunic and basal plate of the onions were removed. The seeds of the apples and pumpkins were removed by cutting out their core. In the case of the carrots, the upper part was cut off as only the storage root was used. The tissues were cut into slices of approximately 5 mm thickness. The slices were put next to each other and vacuum-packed in a plastic bag before blanching at 95 °C for 8 min [26]. Immediately after blanching, the bag was immersed in an ice bath. The slices were frozen with liquid nitrogen and stored at −40 °C until further use.

### 2.3. Generation of the Alcohol-Insoluble Residues and Acid-Unextractable Fractions

The preparation of the AIR was the same for all the matrices and based on the method of McFeeters and Armstrong [27]. After the sliced tissue was thawed, 192 mL technical ethanol was added to 60 g of tissue and mixed (Mixer B-400, Büchi, Flawil, Germany) three times for six seconds. The precipitate was obtained by vacuum filtration (filter paper MN615, Macherey-Nagel, Düren, Germany). Two precipitates were combined for the next mixing step (again three times for six seconds) in 192 mL technical ethanol (Mixer B-400, Büchi, Flawil, Germany). The precipitate, obtained by vacuum filtration, was dispersed in 192 mL technical acetone by magnetic stirring for 10 min. After vacuum filtration, the precipitate was dried overnight at 40 °C to obtain dry AIR. The AIR was grounded with a mortar and pestle and stored in a well-closed container.

The generation of the AcUF was based on the method described by Willemsen et al. [24]. First, 60 g of the AIR was added to 4 L of deionized water at 80 °C. The dispersion was stirred for 30 min (at 80 °C), whereafter the pH was adjusted to 1.6 using 7 M HNO_3_. After extraction for 1 h at 80 °C under continuous stirring, the mixture was cooled down for at least 1 h in an ice bath. Next, the dispersion was centrifuged at 8000× *g* for 10 min (4-16KS, Sigma, Osterode am Harz, Germany) to separate the supernatant and the pellet. The supernatant, which contained the acid extractable fraction, was decanted and the pellet, which contained the AcUF, was washed with 1 L of deionized water. For this, the pellet was resuspended in the water and vacuum filtered over a filter paper (MN615, Macherey-Nagel, Düren, Germany) to reduce the water content of the AcUF. The AcUF (in wet state) was frozen with liquid nitrogen and stored at −40 °C until further use.

### 2.4. Chemical Characterization

The chemical characterization of the AcUF was performed after lyophilization (Alpha 2-4 LSC plus, Christ, Osterode am Harz, Germany).

#### 2.4.1. Analysis of the Uronic Acid Content of the Alcohol-Insoluble Residues and Acid-Unextractable Fractions

First, the AIR or the lyophilized AcUF was hydrolyzed (in duplicate) based on the method of Ahmed and Labavitch [28]. Briefly, 10 mg of the AIR or AcUF was hydrated overnight in 2 mL of deionized water under continuous stirring. Next, 8 mL of sulfuric acid (95% *w*/*w*) was added while the hydrated AIR or AcUF was kept in an ice bath. After 5 min, an additional 2 mL of deionized water was added in a dropwise manner. After 1 h of hydrolysis under continuous stirring in an ice bath, the hydrolysate was diluted to 50 mL with deionized water.

Second, the uronic acid (UA) content was determined based on the spectrophotometric method of Blumenkrantz and Asboe-Hansen [29], using external standards of galacturonic acid. Briefly, 3.6 mL of sulfuric acid (95% *w*/*w*) with sodium tetraborate (0.0125 M) was added to 0.6 mL of the diluted hydrolysate. This mixture was heated at 100 °C for 5 min and cooled down in an ice bath. Next, 60 µL of 0.15% (*w*/*v*) m-hydroxydiphenyl in 0.5% (*w*/*v*) NaOH was added and vortexed for 1 min. After a waiting time of 1 min, the absorbance was measured at 520 nm (Spectrophotometer Genesys 30 Vis, Thermo Fisher, Waltham, MA, USA). A blank was included, meaning that 60 µL of 0.15% (*w*/*v*) m-hydroxydiphenyl in 0.5% (*w*/*v*) NaOH was replaced by 60 µL of NaOH 0.5% (*w*/*v*).

#### 2.4.2. Analysis of the Neutral Monosaccharide Composition of the Acid-Unextractable Fractions

The hydrolysis of the lyophilized AcUF was performed according to the method of Yeats et al. [30] and the determination of the neutral monosaccharide content was executed as described by Van Audenhove et al. [31] (except for the centrifugation step after the hydrolysis which was not performed in the current study). To hydrolyze all polysaccharides present, a Saeman hydrolysis was performed (in duplicate). First, 150 µL of sulfuric acid (72% *w*/*w*) was added to 3 mg of the AcUF and the mixture was vortexed. This mixture was incubated for 1 h at 30 °C and vortexed every 10 min. Second, 4200 µL of ultrapure water was added to reach 4% (*w*/*v*) of sulfuric acid and the sample was hydrolyzed for 1 h at 121 °C. After hydrolysis, the hydrolysate was cooled down in an ice bath, neutralized with 50% (*w*/*w*) NaOH and diluted to 10 mL with ultrapure water. The diluted hydrolysates were stored at −40 °C until further analysis.

High-performance anion exchange chromatography with pulsed amperometric detection was used to determine the neutral monosaccharide content. For this analysis, a Dionex system (ICS-6000, Sunnyvale, CA, USA) was used, equipped with a CarboPac^TM^ PA20 guard column, a CarboPac^TM^ PA20 analytical column and an ED50 electrochemical detector (Dionex, Sunnyvale, CA, USA). If needed, the diluted hydrolysate was further diluted with ultrapure water. Before injection, the diluted hydrolysate was filtered (0.20 µm pore size, Chromafil, Macherey-Nagel, Düren, Germany). To achieve a good separation of all peaks, two different elution profiles at 30 °C were used at a flow rate of 0.4 mL/min. After injection of 10 µL of the diluted hydrolysate, the sample was eluted with 18 mM NaOH for 15 min to separate rhamnose (Rha) and arabinose (Ara), and eluted with 2 mM NaOH for 25 min to separate fucose (Fuc), galactose (Gal), glucose (Glc), xylose (Xyl) and mannose (Man). Both elution profiles were preceded by an equilibration step of 10 min at the respective molarity of NaOH and followed by a washing step of 10 min with 500 mM NaOH. A mixture of monosaccharide standards (L-Fuc, L-Rha, L-Ara, D-Gal, D-Glc, D-Xyl and D-Man) was used as external standard (1–6 ppm) for identification and quantification of the monosaccharides in the diluted hydrolysates. The standard mixture was subjected to the hydrolysis conditions (4% *w*/*v* H_2_SO_4_, 121 °C, 1 h) at different concentrations (1–6 ppm) to calculate a correction factor for each monosaccharide. These correction factors were used to correct for any degradation of the monosaccharides during the hydrolysis.

#### 2.4.3. Analysis of the Degree of Methyl Esterification of the Acid-Unextractable Fractions

The degree of methyl esterification (DM) was determined by Fourier transform infrared spectroscopy based on the method of Kyomugasho et al. [32]. Briefly, the lyophilized AcUF was resuspended in deionized water and adjusted to a pH value of 6.2–6.4 with 0.1 M NaOH. To remove the salts, this suspension was transferred into a dialysis tube and dialyzed in deionized water, which was often refreshed. After dialysis, the dispersion was lyophilized. The lyophilized AcUF was compacted on the sample holder and the infrared spectrum (from 4000 to 400 cm^−1^ with resolution 4 cm^−1^) was obtained (FT-IR spectrophotometer, IRAffinity-1, Shimadzu, Tokyo, Japan) (in duplicate). To correct for possible interference by the proteins present, the spectrum was deconvoluted. From this deconvoluted spectrum, the absorption intensity at 1600 cm^−1^ (COO- stretch) and 1740 cm^−1^ (C=O stretch) was used to calculate the DM using the calibration curve developed by Kyomugasho et al. [32].

### 2.5. Preparation of the Suspensions of the Acid-Unextractable Fractions and High-Pressure Homogenization

The determination of the dry-matter content using a vacuum oven (UniEquip, Planegg, Germany) and the suspension preparation were performed as described by Van Audenhove et al. [31]. Briefly, based on the dry-matter content of the wet AcUF, deionized water was added to obtain a final concentration of 2% (*w*/*w*). The pH value of the suspension was adjusted to 4.5 using 2 M Na_2_CO_3_. The suspension was stored overnight at room temperature to assure complete hydration of the AcUF. After overnight storage, the suspension was mixed for 10 min at 8000 rpm (Ultra Turrax T25, IKA, Staufen, Germany). Part of this suspension was kept aside as non-high-pressure homogenized suspension and denoted as 0 MPa. Part of the remainder of the suspension was high-pressure homogenized (Panda 2k, GEA Niro Soavi, Parma, Italy) at 20 MPa and the other part at 80 MPa. The suspension preparation and HPH treatment were performed in duplicate.

### 2.6. Characterization of the Microstructural Properties of the Acid-Unextractable Fractions in Suspension

#### 2.6.1. Microscopic Visualization

The microstructure of the suspension was visualized by light microscopy in differential interference contrast mode at magnification 10x (Olympus BX-51, Olympus Optical Co. Ltd., Tokyo, Japan) equipped with an XC-50 digital camera [24]. The 2% (*w*/*w*) suspension was diluted as this allowed a clearer observation of the microstructural properties of the single entities present in the suspension. Therefore, a 0.6% (*w*/*w*) suspension was prepared by diluting the 2% (*w*/*w*) suspension with deionized water adjusted to a pH value of 4.5 with 0.1 M HCl, followed by magnetic stirring at room temperature for at least 1 h. Briefly, 100 µL of each (diluted) suspension was placed on a microscope slide and around 10 images were taken, of which a representative micrograph was chosen.

#### 2.6.2. Particle Size Distribution Analysis

The volume-based particle size distribution (PSD) of the particles present in the suspension was determined by laser diffraction (Laser Diffraction Particle Size Analyzer, LS 13 320, Beckman Coulter Inc., Indianapolis, IN, USA). As described in more detail by Verkempinck et al. [33], the sample was dispersed in a tank filled with deionized water (to obtain an obscuration around 8-10%) and the scattering and intensity of the diffracted laser light was analyzed by the Fraunhofer model to obtain the PSD over the range from 0.04 to 2000 µm. In order to facilitate a fast comparison of the mean particle sizes of the different suspensions, the mean volume-based particle size (d_4,3_) was calculated. Two runs were performed in sequence (each taking 90 s at pump speed 30%); however, to avoid any interference due to weak aggregation, only the second run was taken into account to calculate the PSD. This analysis was performed in duplicate by loading each suspension twice.

### 2.7. Characterization of the Functional Properties of the Acid-Unextractable Fractions in Suspension

#### 2.7.1. Rheological Analysis

Relevant viscoelastic properties of the suspension were determined by oscillatory rheological analysis performed with a stress-controlled rheometer (MCR 302, Anton Paar, Graz, Austria). The analysis was based on the method of Willemsen et al. [34] and was performed with the concentric cylinder system (2 mm gap), with rough surfaces designed and used by Willemsen et al. [34]. After loading the suspension, a pre-shear was performed for 1 min at 10 s^−1^, followed by 1 min of rest. Next, the suspension was kept for 3 min at an oscillatory regime with a constant angular frequency of 10 rad/s and 0.1% strain. In order to gain insight into the relation between the viscoelastic properties of the suspension (mainly the G′, loss modulus (G″) and loss factor) and the angular frequency, a frequency sweep was performed from 100 to 0.1 rad/s (logarithmically decreasing, 7 points per decade) at a constant strain of 0.1%. Afterwards, for 1 min, the suspension was kept again at a constant angular frequency of 10 rad/s and 0.1% strain. As a final step, a strain sweep was performed from 0.01% to 100% strain (logarithmically increasing, 7 points per decade) at constant angular frequency of 10 rad/s to be able to determine the critical strain of each suspension. In the current study, the critical strain, quantifying the limit of the linear viscoelastic region, was defined as the strain at which the G′ was decreased to 90% of its plateau value at low strain and was calculated by interpolation. The constant strain of 0.1% during the frequency sweep was chosen based on preliminary tests, and was validated to be within the linear viscoelastic region using the data of the strain sweep. These different rheological tests were performed in sequence. The analysis was carried out in duplicate by independent sample loadings.

#### 2.7.2. Measurement of Water Binding Capacity

The WBC of the AcUF was measured by performing centrifugation in suspended state [35]. The suspension was centrifuged at 1000× *g* for 30 min at 25 °C (LUMiFuge, LUM GmbH, Berlin, Germany). Based on the concentration of the AcUF in suspension (i.e., 2% *w*/*w*), the exact mass of the suspension added to the polycarbonate cuvette and the mass of the supernatant obtained after centrifugation, the WBC was calculated using Equation (1). This analysis was performed in duplicate for each suspension.

(1) $$\mathrm{WBC}=\frac{\mathrm{mass}\;\mathrm{suspension}\cdot 0.98-\mathrm{mass}\;\mathrm{of}\;\mathrm{supernatant}}{\mathrm{mass}\;\mathrm{suspension}\cdot 0.02}$$

where WBC is the water binding capacity (g water/g).

### 2.8. Statistical Analysis

For the chemical characterization of the AcUF, a sample was taken from two independent extractions. These two samples of each AcUF or one sample of the AIR batch were analyzed twice. The preparation of all suspensions was carried out twice; on each suspension, all analyses were performed in duplicate. On these four values (i.e., *n* = 2 × 2) or two for the UA content determination of the AIR (i.e., *n* = 2), the average and standard deviation were calculated. The significances of the differences between means were assessed using Tukey’s range test (*p* < 0.05).

## 3. Results and Discussion

### 3.1. Chemical Characterization

**Figure 1:**
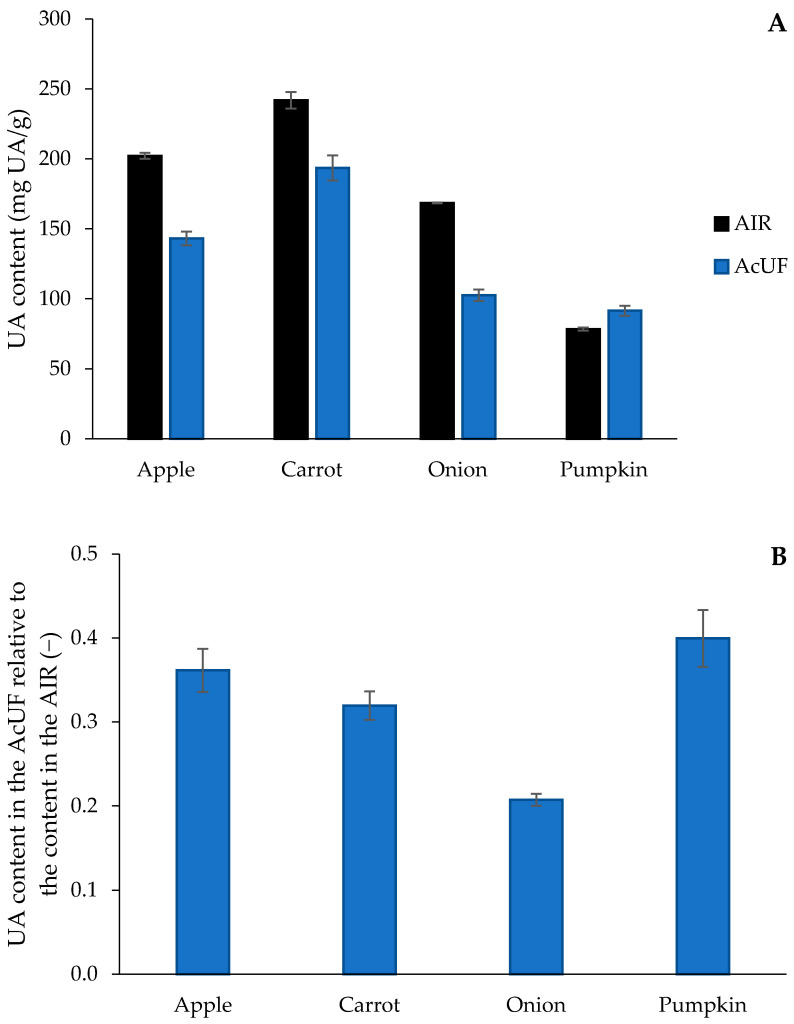
Uronic acid (UA) content in the alcohol-insoluble residues (AIR) (*n* = 2) and the acid-unextractable fractions (AcUF) (*n* = 2 × 2) of the different matrices (**A**) and the UA content in the AcUF expressed relatively to the content in the AIR (**B**). The error bars represent the standard deviation.

Between the matrices studied, substantial differences in the UA content of the AIR were observed (Figure 1A). The UA content in the AIR of apple was 202 mg/g, which is in the range of what was observed by Renard et al. [36] (270 mg/g), Massiot et al. [37] (287 mg/g) and Cybulska et al. [38] (around 150 mg/g). The highest amount of UA was found in the AIR of carrot (242 mg/g), which is in good agreement with Houben et al. [39] (239 mg/g). The UA content of 168 mg/g in the AIR of onion is lower than reported before by Redgwell and Selvendran [40] (279 mg/g) for onion CWM obtained after extensive purification. The lowest UA content was observed for the AIR of pumpkin (78 mg/g), which is lower than what was observed by Atencio et al. [23] for the AIR of pumpkin pomace (136 mg/g). In Figure 1B, the retention of UA in the AcUF expressed to the total amount in the AIR is shown. The most efficient extraction of pectin (based on the UA content) was reached for onion, whereas the lowest pectin extraction efficiency was observed for pumpkin. The UA content was even higher in the AcUF than in the AIR of pumpkin. This can be attributed to the high starch and protein content in the pumpkin AIR [23,41], which is expected to be co-extracted with pectin during the acid extraction. In this context, de Escalada Pla et al. [41] observed co-extraction of a high amount of Glc, partially originating from starch, during cold water pectin extraction from pumpkin.

In order to gain insight into the polysaccharide composition of the AcUFs, the monosaccharide content after Saeman hydrolysis was analyzed (Table 1). Although an acid extraction was performed on the AIR to obtain the AcUF, a substantial amount of UA was still observed, which ranged between 12.5 and 25.9 mol%. It has been shown before for lemon peel CWM [24] and tomato CWM [31] that a single-step nitric acid extraction was less efficient in extracting pectin than a stepwise approach using hot water, a chelating agent and a low-alkaline medium, in which specific pectin fractions are extracted [42]. The most abundant monosaccharide was Glc, which ranged between 55.2 and 69.5 mol%, indicating a high cellulose and hemicellulose content. Regarding fruits and vegetables, the most prevalent hemicelluloses in dicotyledonous plants (such as carrot, apple and pumpkin) and monocotyledonous plants (such as onion) are xyloglucan (in particular, in dicots), xylan (in particular, in monocots) and, in minor amounts, glucomannans [3,43], which explains the presence of Xyl (3.0 mol% in carrot to 10.6 mol% in apple) and Man (2.5 mol% in pumpkin to 3.7 mol% in carrot) in the AcUFs of the different matrices. Part of the Xyl could also originate from the xylogalacturonan region in the residual pectin; particularly apple pectin contains a substantial amount of xylogalacturonan [44,45,46]. Nevertheless, based on the results of Renard et al. [36], it is clear that the main part of Xyl in apple CWM could be attributed to the hemicellulose fraction. Since both apple [36,37,44] and carrot [39,43,44,47,48] have rather branched pectin, implying a relatively high amount of Ara and Gal, the high amounts of Ara and Gal in the apple and carrot AcUF could mainly be attributed to the residual pectin. A high amount of Gal was also found in the AcUF of onion and pumpkin, together with a relatively low amount of Ara. In the onion AcUF, the high content of Gal could be attributed to the high abundance of the Gal-rich side chains in the residual pectin (in which only a small amount of Ara is reported) [49] and the presence of galactans, which are not linked to pectin [50,51,52]. Kurz et al. [45] reported a higher amount of Gal than Ara in the AIR of Hokkaido pumpkin, which is in accordance with the current results. However, one should note that the pectin molecule could be partially hydrolyzed during the acid pectin extraction to which especially the side-chains, in particular the arabinan side-chains, are prone [53,54]. Therefore, the amount of Ara and Gal in the more strongly bound pectin fraction (as it was not acid extractable), and thus, its extent of branching could be higher in the native form than reported here (as part of the AcUF).

The highest DM (Table 1) was observed for the pumpkin AcUF (53.4%). The DM of the apple, carrot and onion AcUF was low and very similar (37.0–40.4%). This value is lower than what has been reported before for the AIR of apple (72%) [36] and carrot (67%) [39], whereas, for the AIR of fresh onions, comparable values (30–46%) to the onion AcUF in the current study were reported [55]. In contrast to the AIR, the DM of the AcUF only results from the DM of the residual pectin, and thus, the pectin fraction, which was more strongly interacting in the cell wall, instead of from the total pectin fraction in the respective matrix. Furthermore, during the acid extraction, a certain extent of demethoxylation could occur due to the low pH value (i.e., 1.6) and the high temperature (80 °C) [31,56].

The contribution of the pectin backbone (i.e., UA + Rha) (mol%) [31] and the ratio of Glc to UA (−) was calculated to facilitate an easy and fast comparison of the compositional properties of the AcUFs of the different matrices (Table 1). The contribution of the pectin backbone to the polysaccharides was clearly higher for the carrot AcUF than for the other matrices. Moreover, large differences in the molar ratio of Glc to UA were observed. More specifically, the ratio of cellulose and Glc-containing hemicelluloses to pectin (estimated by the UA content) in the AcUF decreased in the following order: pumpkin, onion, apple and carrot. Based on this compositional analysis, it is clear that not only the residual pectin content but also the relative contribution of non-pectin polysaccharides to pectin was highly differing among the matrices studied.

### 3.2. Microstructural Characterization

#### 3.2.1. Microstructure of the Acid-Unextractable Fractions in Suspension before High-Pressure Homogenization

**Figure 2:**
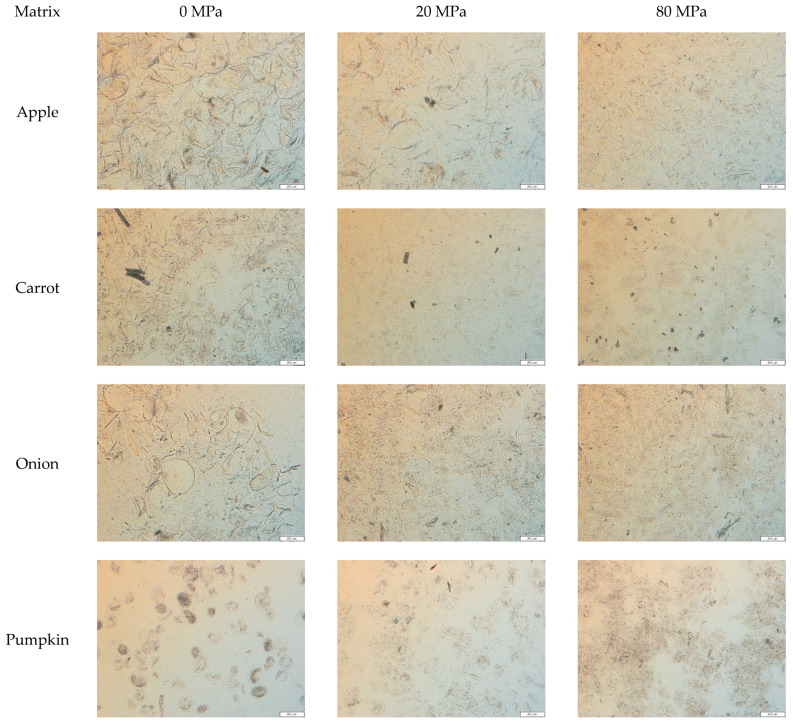
Microscopic visualization of the suspensions prepared with the acid-unextractable fraction of apple, carrot, onion and pumpkin at 0.6% (*w*/*w*) concentration before (0 MPa) and after high-pressure homogenization at 20 and 80 MPa. The scale bar represents 200 µm.

The microstructure of the AcUF suspensions at 0.6% (*w*/*w*) concentration of the different matrices is visualized in Figure 2. For all the matrices studied, the particles in the AcUF still showed a cell-like morphology, even though 60 to 80% of the UA in the AIR was removed by the acid extraction (Figure 1B). Thus, partial pectin depletion of the cell wall network of these matrices did not result in a complete loss of the cell wall intactness. For the CWM of apple, Vetter and Kunzek [57] also observed that the original cell shape was retained after (partial) removal of pectin by extraction. In the apple, onion and pumpkin AcUF, the particles with cell-like morphology occurred separately, which is the result of the solubilization of pectin during the ripening and/or high-temperature blanching and the (partial) removal of the middle lamella pectin, responsible for cell adhesion by the acid pectin extraction [10,58]. In a study of Yi et al. [59] on non-thermally treated apple juice, the CWM was present both as single cells and as cell clusters before HPH, which highlights again the role of the blanching step and the pectin depletion applied on the cell–cell adhesion.

In contrast, the carrot AcUF was mainly constituted by particles with intact cell-like morphology occurring in a clustered way. Other researchers, who performed high-temperature blanching on carrot tissue, namely 5 min at 95 °C [60] and 40 min at 90 °C [61], also observed the presence of cell clusters after blending. However, when more intense conditions were applied, namely 30 min at 100 °C followed by mixing for 8 min, mainly single cells were observed [62]. In the current study, the observation of these clusters in carrot is remarkable since not only high-temperature blanching (8 min at 95 °C) but also acid pectin extraction was performed on the carrot tissue during the preparation of the AcUF, resulting in a 68% removal of the UA present in the carrot AIR. As the intact partially pectin-depleted cell walls occurred in a clustered manner, it is clear that the ability of the middle lamellae in the carrot CWM to keep the adjacent cells together was not impaired by the loss of a high amount of pectin. In this context, in microscopic visualizations of a suspension of both tomato and high-temperature blanched (5 min at 95 °C) carrot tissue, clusters of carrot cells were still visible after incubation, allowing the endogenous tomato pectinases to (partially) break down the pectin present [63].

In principle, the particles in the AcUF of apple, onion and pumpkin are comparable to “*single cells*” and in the AcUF of carrot to “*cell clusters*” in regular fruit and vegetable-based suspensions. However, since, in the current study, only the CWM was retained in the AcUF, this description is not used here.

**Figure 3:**
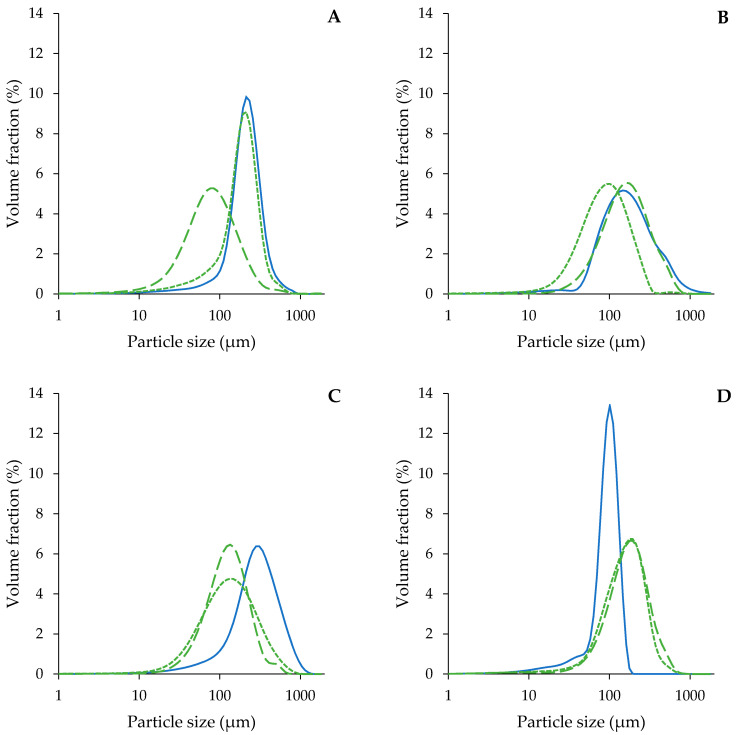
Volume-based particle size distribution of the suspensions prepared with the acid-unextractable fraction of apple (**A**), carrot (**B**), onion (**C**) and pumpkin (**D**) before (solid blue line) and after high-pressure homogenization at 20 MPa (green short dashed line) and 80 MPa (green long dashed line).

**Figure 4:**
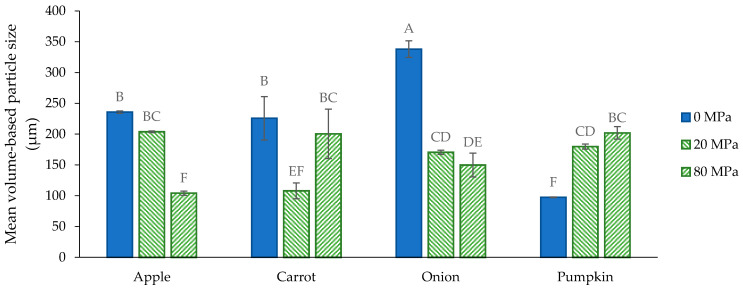
Mean volume-based particle size (d_4,3_) of the suspensions prepared with the acid-unextractable fraction of apple, carrot, onion and pumpkin before (0 MPa) and after high-pressure homogenization at 20 and 80 MPa. The error bars represent the standard deviation (*n* = 2 × 2) and significant differences are indicated by different letters (Tukey test, *p* < 0.05).

Visually, the size of the cellular structures of the apple, onion and carrot AcUF was clearly larger than for the pumpkin AcUF. This was supported by the PSDs (Figure 3) and the differences in the d_4,3_ (Figure 4). Apart from a shoulder at large particle size in the case of the carrot AcUF, all PSDs showed a monomodal distribution. The particles in the onion AcUF suspension had the highest d_4,3_ and the broadest size distribution among the matrices showing no clusters in their AcUF, which could also be observed in the micrographs (Figure 2). In the literature, a broad range of onion cell size was also reported, ranging from 200 × 250 to 370 × 500 µm [64]. The d_4,3_ of the apple AcUF was 236 µm and is comparable to other research reporting that the peak of the PSD of single apple cells was around 180–200 µm [12,65]. The pumpkin AcUF was characterized by very small and spherical particles in comparison to the other matrices. The d_4,3_ of these particles was 97 µm, which is in accordance with Atencio et al. [23] who observed that the peak of the PSD in the AcUF of pumpkin pomace was approximately 100 µm and to Mayor et al. [66], who reported a mean diameter for pumpkin cells of 136 µm. The particles in the carrot AcUF suspension had a d_4,3_ of 226 µm and varied greatly in particle size. The PSD of the carrot AcUF suspension showed that around 90% of the particles had a diameter larger than the mean diameter of carrot single cells (i.e., 60–70 µm) [13,62]. As a consequence, it is clear that, in line with the respective micrographs, most of the particles were clusters with a high variation in the number of the constituting elements.

#### 3.2.2. Microstructure of the Acid-Unextractable Fractions in Suspension after High-Pressure Homogenization and the Effect of Pressure Level

The AcUF suspensions were high-pressure homogenized at 20 and 80 MPa. Although the particles in the AcUF of apple, onion and pumpkin all consisted mainly of intact partially pectin-depleted cell walls, the effect of high shear by HPH was clearly different. In the case of the apple AcUF, applying HPH at 20 MPa had only a very limited effect on the microstructure of the suspension. Indeed, in the micrographs (Figure 2), it is visible that the effect of HPH was limited to a slight deformation of the partially pectin-depleted cell walls instead of disruption. A pressure level of 20 MPa was thus not sufficient to overcome the strength of the partially pectin-depleted cell walls of apple. By HPH at 80 MPa, the disruption of the AcUF was clearly more prominent. These observations were also reflected in the PSD (Figure 3A). Whereas the d_4,3_ was only decreased from 236 to 204 µm by HPH at 20 MPa, the d_4,3_ was reduced to 104 µm after HPH at 80 MPa (Figure 4). It was also shown by Yi et al. [59] that HPH of apple juice at 20 MPa resulted in a d_4,3_ being still slightly above 200 µm, which was further reduced when applying higher pressure levels. In the current work, even after HPH at 80 MPa, the CWM was still mainly occurring as broken cell wall structures instead of as highly disrupted CWM.

The strength of the partially pectin-depleted cell walls of onion seemed lower than those of apple, as the cell-like morphology was already completely lost by HPH at 20 MPa. In the micrographs, broken cell wall structures were observed together with more disrupted CWM (Figure 2). Furthermore, upon HPH at 80 MPa, a more highly disrupted AcUF and less cell wall structures were observed. This gradual particle disruption with increasing pressure level was also reflected in the decreasing particle size from 338 (0 MPa) to 170 (20 MPa) to 150 µm (80 MPa).

In the case of the pumpkin AcUF, almost all of the particles with intact cell-like morphology were disrupted both by HPH at 20 and 80 MPa. However, the presence of clumps of disrupted CWM in the pumpkin AcUF after HPH at 20 and 80 MPa suggests a stronger aggregation than for the AcUF of apple and onion, of which the disrupted material occurred more as a continuum. This aggregation is also reflected by the increase in the d_4,3_ by HPH at 20 MPa from 97 to 180 µm and to 202 µm by HPH at 80 MPa. This aggregation upon disruption of the cell wall network might (partially) be the result of the interaction between the residual protein fraction and the polysaccharides, e.g., by the formation of coacervates between proteins and pectin [67,68], as it has been shown that the protein concentration in the AcUF is high [23]. The observed increase in the d_4,3_ with pressure level is in contrast to what was observed by Atencio et al. [23] for a suspension of the AcUF of pumpkin pomace, for which a decrease in the upper limit of the 90% smallest particles was observed with increasing pressure level.

The most remarkable relation between the microstructure of the AcUF suspension and the intensity of the applied shear was observed for the carrot AcUF. HPH at 20 MPa resulted in the breakdown of the clusters; however, next to particles having the dimensions of an intact single cell, broken cell wall structures were also observed. After HPH at 80 MPa, interestingly, the more extensively disrupted AcUF occurred in a clumped manner. It thus seemed that when the CWM was sufficiently released and opened, aggregation between the cell wall remnants was possible. This peculiar behavior between the intensity of the applied shear by HPH and the resulting microstructure is also reflected in the PSD. While the d_4,3_ was lower after HPH at 20 MPa (108 µm) than without HPH (226 µm) due to the breakdown of the clusters, the d_4,3_ after HPH at 80 MPa (200 µm) was much higher than after 20 MPa, attributed to the existence of rather stable aggregates. In other work on carrot-based suspensions [1], it was also described that HPH led to the disruption of the carrot cell clusters to smaller clusters, single cells and cell fragments. A larger particle size after HPH at 80 MPa than before HPH was also observed by Sankaran et al. [69] for a carrot suspension which was incubated with a pure pectinase for 8 h. This higher particle size was attributed to the wooly-like structure obtained after HPH instead of dispersed cell wall fragments [69].

In the high-pressure homogenized suspensions, the microstructural attributes of the cell walls, as visualized in the micrographs, can be described, in general terms, by three levels of intactness, namely slightly deformed intact cell walls, broken cell wall structures and extensively disrupted CWM remnants being present as a continuum or in clumps due to aggregation. The broken cell wall structures are likely formed when the impact of the applied shear by HPH was sufficient to break a substantial fraction of the cell walls at their weakest spot(s), but too low to extensively disrupt the AcUF, which would result in a continuum (with a certain extent of aggregation) of CWM remnants. The susceptibility of pectin-depleted cell walls to high shear was clearly dependent on the botanical origin, which was evaluated by the observed differences in extent of disruption of the AcUF (Figure 2). The observation that, for a certain matrix at a certain pressure level, microstructural attributes indicating different levels of CWM intactness occurred simultaneously suggests the existence of variability in the strength of the pectin-depleted cell walls originating from the same matrix.

### 3.3. Functional Characterization

#### 3.3.1. General Considerations

**Figure 5:**
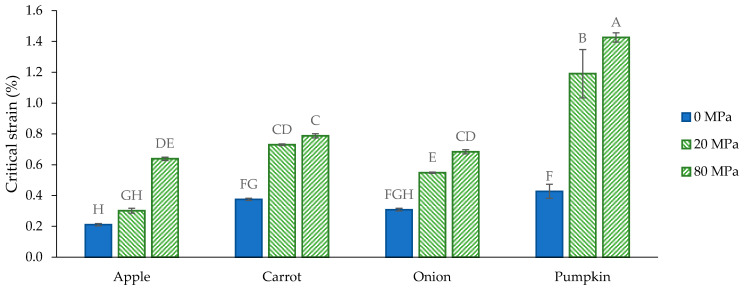
Critical strain of the suspensions (2% *w*/*w*) prepared with the acid-unextractable fraction of apple, carrot, onion and pumpkin before (0 MPa) and after high-pressure homogenization at 20 and 80 MPa at 10 rad/s angular frequency; data are from the strain sweep. The error bars represent the standard deviation (*n* = 2 × 2) and significant differences are indicated by different letters (Tukey test, *p* < 0.05).

**Figure 6:**
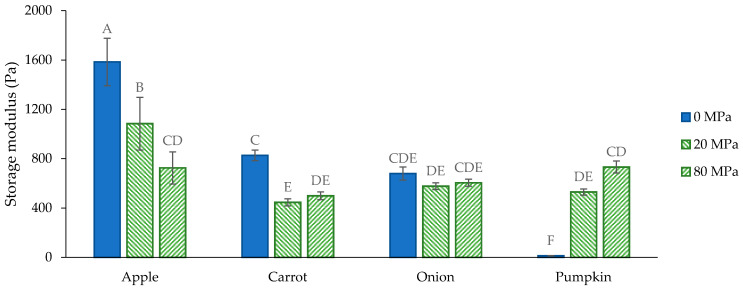
Storage modulus (G′) of the suspensions (2% *w*/*w*) prepared with the acid-unextractable fraction of apple, carrot, onion and pumpkin before (0 MPa) and after high-pressure homogenization at 20 and 80 MPa at 10 rad/s angular frequency and 0.1% strain; data are from the frequency sweep. The error bars represent the standard deviation (*n* = 2 × 2) and significant differences are indicated by different letters (Tukey test, *p* < 0.05).

**Figure A1:**
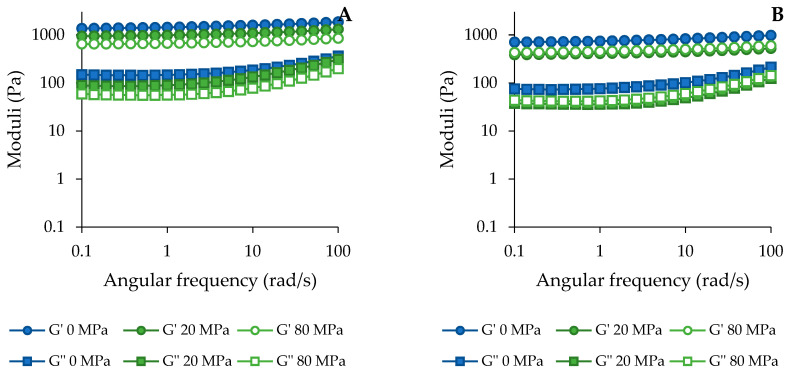

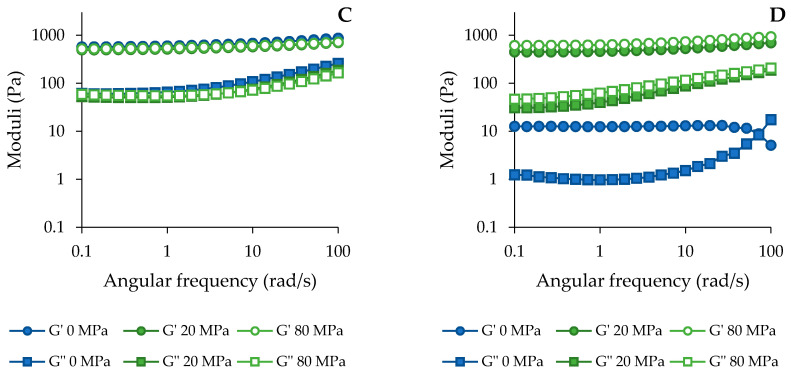
Storage modulus (G′) and loss modulus (G″) in function of the angular frequency (frequency sweep) at strain 0.1% of the suspensions prepared with the acid-unextractable fraction of apple (**A**), carrot (**B**), onion (**C**) and pumpkin (**D**) before (0 MPa) and after high-pressure homogenization at 20 and 80 MPa.

The relation between the angular frequency and the G′ and G″ at strain 0.1% is shown in Figure A1. For all suspensions, this frequency sweep was performed in the linear viscoelastic region as the critical strain was clearly above 0.1% for each suspension (Figure 5). The results of the frequency sweeps of the AcUF suspensions of the different matrices are comparable to what was found for other fruit- and vegetable-based suspensions [57,62,70,71,72,73,74,75]. The AcUF of all the matrices was able to form an elastic gel-like network in the suspensions, as the loss factor ranged from 0.11 to 0.17 (assessed at angular frequency 10 rad/s and strain 0.1%) [76]. Based on the low dependency of the G′ on the angular frequency (Figure A1) and the rather low critical strain (Figure 5), the suspensions can be classified as “weak physical gels” [77,78,79]. Interactions occurring in the CWM suspensions are mainly hydrogen bonding, van der Waals forces, hydrophobic interactions and electrostatic interactions [78,79]. Overall, the results of the frequency sweeps from all matrices seemed very similar. Therefore, for ease of comparison, in what follows, the G′ will be compared between the different suspensions at a fixed angular frequency (namely, 10 rad/s) and at strain 0.1% (Figure 6) as a measure of the stiffness to assess the ability of the AcUF to build a network in suspension and how this is affected by HPH at 20 and 80 MPa [24,80].

#### 3.3.2. Functional Properties of the Acid-Unextractable Fractions in Suspension before High-Pressure Homogenization

**Figure 7:**
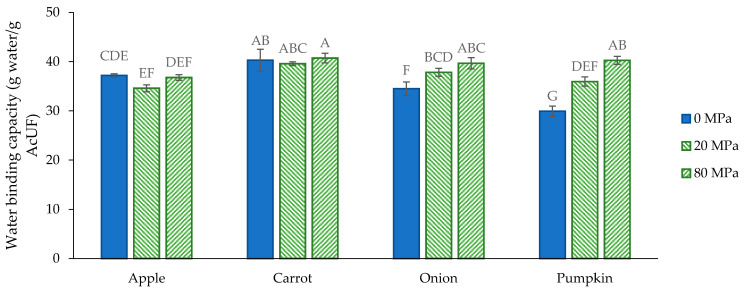
Water binding capacity (2% *w*/*w*) of the acid-unextractable fraction of apple, carrot, onion and pumpkin before (0 MPa) and after high-pressure homogenization at 20 and 80 MPa. The error bars represent the standard deviation (*n* = 2 × 2) and significant differences are indicated by different letters (Tukey test, *p* < 0.05).

The WBC of the AcUFs in suspension at 2% (*w*/*w*) ranged from 30 g water/g for the pumpkin AcUF to 40 g water/g for the carrot AcUF (Figure 7). Since the AcUF of all matrices still showed a cell-like morphology, it is expected that the water was not only interacting by ionic and hydrogen bonds and hydrophobic interactions with the surface of the particles or within capillaries and voids [81,82,83,84,85], but also physically entrapped within the cell wall borders. A certain relation seemed to exist between the WBC and the absolute UA content (as an estimation of the pectin content) in the AcUF. Indeed, the same sequence of the AcUFs of the matrices studied was obtained when ranked in increasing order of the WBC, as when ranked in increasing order of absolute UA content (i.e., pumpkin < onion < apple < carrot).

The role of pectin in the AcUF suspensions should be further discussed. On the one hand, pectin has an intrinsic potential to bind water, which is mainly related to the presence of hydrophilic groups, such as hydroxyl and carboxyl groups [86]. On the other hand, the removal of pectin from the cell wall structure likely created an additional amount of voids, which could result in a higher potential to bind water [23,24]. The differences in the retention of UA (Figure 1B) in particular could be involved in the substantially higher WBC of the onion AcUF compared to the pumpkin AcUF, of which the absolute UA contents were very similar (103 and 91 mg UA/g, respectively). Since the retention of UA after extraction was clearly different (21% for the onion AcUF and 40% for the pumpkin AcUF), the higher WBC of the onion AcUF than of the pumpkin AcUF could probably be partly linked to the higher removal of pectin, and thus, the higher amount of voids present in the network. Furthermore, it should be noted that the higher DM of the pumpkin AcUF than of the onion AcUF probably also contributed to the lower WBC of the former, as a lower amount of polar groups (higher DM) has a negative effect on the interaction with water [81,87]. Overall, based on this set of matrices, it is suggested that the AcUF of fruits and vegetables with a high pectin content (in the AIR) have a higher WBC, because of the higher absolute residual pectin content, at least if still a considerable fraction (e.g., between 20% and 40%) of the pectin is retained in the AcUF.

Before HPH, a large difference existed between the G′ of the AcUF suspensions of the different matrices. The lowest value was obtained for the pumpkin AcUF suspension (13 Pa) and the highest for the apple AcUF suspension (1585 Pa). For the non-homogenized suspensions, the network was formed by the particles with cell-like morphology (in the case of the apple, onion and pumpkin AcUF) or particles with intact cell-like morphology occurring in a clustered way (in the case of the carrot AcUF). No evident relation seemed to exist between the G′ of the suspensions and the d_4,3_ or the WBC. This is not unexpected. It is very difficult to define the specific effect of the main factors such as the chemical composition (Figure 1 and Table 1), general microstructure (Figure 2), PSD (Figure 3) and WBC (Figure 7) on the G′ (Figure 6), especially when different matrices are compared. Nevertheless, some notable observations are discussed further. A clear effect of the size and shape of the particles could be observed. Indeed, the pumpkin AcUF suspension, which mainly consisted of small and very regular shaped particles, had a much lower G′ than the AcUF suspensions of the other matrices, which consisted of larger particles with irregular shapes. Irregularities (and larger particles) are known to contribute to particle network formation by frictional effects next to packing effects [13,88,89]. Although it is clear that the properties of the particles of the pumpkin AcUF resulted in the low potential to build a network, a large variation in G′ was still observed for the suspensions of the other matrices. The particles of the apple AcUF suspension showed a much lower susceptibility to disruption by HPH at 20 MPa than the particles in the AcUF suspensions of the other matrices (Figure 2), which suggests that the intact cell walls of apple had the highest strength among the matrices studied, but still were sufficiently deformable to pass through the homogenizer without being disrupted. This higher strength of the pectin-depleted cell walls of apple than of carrot and onion was likely an important factor, leading to the clearly higher stiffness of the apple AcUF suspension.

#### 3.3.3. Functional Properties of the Acid-Unextractable Fractions in Suspension after High-Pressure Homogenization and the Effect of Pressure Level

The effect of HPH on the WBC of the AcUF was matrix-dependent (Figure 7). For the apple and carrot AcUF, no significant effect of HPH on the WBC was observed, whereas the WBC of the onion and pumpkin AcUF clearly increased by HPH and with increasing pressure level. After HPH (at both pressure levels), the WBC of the AcUF was not related to the absolute UA content (or the retention) in the AcUF, as was observed before HPH. Nevertheless, since the UA content seemed to be related with the WBC of the non-homogenized AcUF, it may still have an effect on the value of the WBC of the homogenized AcUF because of the different levels of the WBC before HPH. However, it is reasonable that the effect of HPH on the WBC cannot be linked only (or mainly) to one factor, due to the diversity in the microstructural attributes of the AcUF of the matrices studied and the resulting impact of HPH on this microstructure.

For all the matrices, the integrity of the particles with cell-like morphology was lost after HPH, except for the apple AcUF, for which the impact of HPH at 20 MPa was very limited (Figure 2). After HPH, the particles had smaller dimensions, although this was not always reflected in the PSD because of aggregation (Figure 3). Thus, the resulting impact of HPH on the WBC of the AcUF is expected to be the net result of the loss of part of the physically entrapped water on the one hand and an additional amount of water which can be bound on the AcUF after disruption by HPH on the other hand. Indeed, a higher water binding could be expected because of the larger surface of the particles with more binding spots being exposed to the serum phase (as smaller particles are formed) and the possible creation of additional voids by HPH [24,85]. In contrast to the apple and carrot AcUF, a significant increase in the WBC upon HPH was observed for the AcUF of onion and pumpkin. Taking into account that, in general terms, the effect of HPH on the microstructural properties of the onion and pumpkin AcUF was comparable as for the apple and carrot AcUF, this clear increase in the WBC suggests that the disrupted AcUF of onion and pumpkin has a higher potential to swell than the disrupted AcUF of apple and carrot.

Additionally, the effect of HPH on the G′ of the AcUF suspensions was matrix-dependent (Figure 6). Previous studies on the role of HPH on the texturizing potential of the AcUF, prepared from the AIR of lemon peel and pumpkin pomace, reported an increase in the G′ of the suspension by HPH [23,24]. In the set of botanical origins covered by the current study, only the texturizing potential of the pumpkin AcUF was improved by HPH, which is in accordance with Atencio et al. [23]. The texturizing potential of the onion AcUF was unaffected by HPH; meanwhile, for the apple and carrot AcUF, a prominent decrease was observed. In what follows, the effect of HPH on the G′ of the suspensions of the AcUF of the different matrices will be compared with the literature, in particular for apple and carrot. However, it should be noted that most of these studies investigated the rheological properties of suspensions prepared with the whole fruit or vegetable instead of with partially pectin-depleted CWM. More specifically, it is expected that pectin depletion increased the susceptibility of the CWM to breakdown by HPH and the potential to swell because of the voids created in the cell wall network [23,24,69]. In contrast to the effect of HPH on the apple AcUF suspension, Bengtsson and Tornberg [17] observed an increase in the G′ of apple suspensions with an increasing number of passages through a high-pressure homogenizer. However, this discrepancy could also be attributed to the much lower pressure level used (9 MPa) than applied in the current study (20 and 80 MPa), which resulted in a very small effect on the microstructure of the apple suspension [17].

Furthermore, HPH also led to a lower G′ of the carrot AcUF suspension with no significant difference between the treatment at 20 and 80 MPa. Bengtsson and Tornberg [17] observed that at low pressure level (9 MPa) for multiple passages, the cell clusters were only reduced in size and that this led to an increase in the G′ of the suspension. On the other hand, Lopez-Sanchez et al. [1], who applied HPH on a carrot suspension at higher pressure level (60 MPa) resulting in small cell clusters and a high fraction of fragments, observed that the G′ of the homogenized suspension was clearly lower than the G′ of the non-homogenized suspension. In accordance with the impact of HPH on the texturizing potential of pectin-depleted CWM of carrot, also in other studies [14,62], it was shown that the potential of carrot tissue to build a stiff network in suspension decreased when the particles were broken down from cell clusters to single cells to cell fragments.

Thus, in the case of both the apple and carrot AcUF, the network formed by the large particles seemed to have a higher stiffness than the network formed by the cell wall fragments. The large difference between the G′ of the apple suspensions homogenized at 20 and 80 MPa is in accordance with the clearly higher impact of HPH at 80 MPa on the microstructure. On the other hand, the small difference between HPH at 20 and 80 MPa on the G′ of the carrot AcUF suspension implies that the texturizing potential was reduced by the breakdown of the clusters and of most of the particles constituting the clusters but not by further degradation. In the case of the onion AcUF, the G′ was unchanged by HPH, indicating that the texturizing potential of the particles with intact cell-like morphology was similar to the texturizing potential of the cell wall fragments. In the case of the pumpkin AcUF specifically, HPH, which resulted in an efficient release and opening of the pectin-depleted cell walls, was an excellent approach to functionalize the pectin-depleted CWM of pumpkin.

It has been shown before that a higher interaction with water is related to a higher G′ of the suspension [1,23,90]. However, in the current study, no relation between the WBC and the G′ was observed at any of the pressure levels of HPH for all matrices, which is probably, again, related to the high diversity in properties of the AcUFs originating from different botanical origins. In the context of the effect of HPH on the texturizing potential of the AcUFs, it should be noted that, as discussed above, a significant increase in the WBC with increasing pressure level (and thus, increasing impact by HPH) was observed for the onion and pumpkin AcUF. This higher interaction with water after HPH likely had an important effect on the G′ of the suspension. Indeed, although the disruption caused the network of the intact particles with cell-like morphology to no longer be formed, it probably improved the swelling of the CWM, resulting in a higher phase volume, and thus, a better texturizing potential [1]. This can (partially) explain the non-significant effect on the G′ of the onion AcUF suspension and the tremendous increase in the G′ of the pumpkin AcUF suspension. The fact that the increase in the G′ for the pumpkin AcUF was so prominent is, of course, to a high extent related to the low stiffness of the network formed by the small particles in the non-homogenized pumpkin AcUF suspension.

Despite the considerable differences in the polysaccharide composition of the matrices studied, the variation in the WBC (37 to 41 g water/g) and the G′ (498 to 732 Pa) of the suspensions being high-pressure homogenized at 80 MPa was limited. Although several matrices were compared, one should not generalize the ranges reported here to other botanical origins, e.g., a clearly lower G′ (around 140 Pa at frequency 6.28 rad/s and strain 1%) was obtained for the AcUF of lemon peel (at the same concentration) after HPH at 80 MPa [24]. In this regard, the specific alterations in the assembly of the polymers in the pectin-depleted CWM upon HPH and the possible (consequent) interactions between the CWM and with water have probably also an important effect on the resulting texturizing properties (e.g., the significant increase in the WBC of the AcUF of onion and pumpkin in contrast to the other matrices). For example, Lopez-Sanchez et al. [1] visualized using cryo-scanning electron microscopy that the compact structure of the cell wall of carrot was retained after HPH, but that the cell wall of tomato was swollen, leading to the better texturizing potential of the latter.

To assess the strength of the network, the critical strain was calculated for each suspension [78] (Figure 5). It is clear that a network with a higher strength could be formed by the AcUF after disruption by HPH (at 20 and 80 MPa), probably because of the entanglements, which could be formed between the CWM fragments instead of by stacking of particles (at 0 MPa) [20]. The non-significant increase in the critical strain of the apple AcUF suspension by HPH at 20 MPa can be attributed to the very small effect of this treatment on the microstructure, whereas a clear increase was observed by HPH at 80 MPa, as this microstructure allowed more entanglements. For the other matrices, an increase in shear force by HPH only led to a small increase in the critical strain, which again confirms that the effect of higher pressure level on the texturizing potential of the AcUF of these matrices was limited.

## 4. Conclusions

In the current study, the texturizing potential of the AcUF, which is the unextractable CWM fraction obtained after nitric acid pectin extraction on the AIR, was investigated for different fruits and vegetables, namely apple, carrot, onion and pumpkin. Furthermore, the potential of functionalization of this AcUF by HPH at 20 and 80 MPa was evaluated. After the acid extraction, 20–40% of the UA content present in the AIR (depending on the specific matrix) was retained in the AcUF. The contribution of the pectin backbone to the total polysaccharides in the AcUF decreased from carrot to apple to onion to pumpkin.

The particles present in the AcUFs displayed a cell-like morphology, indicating that some intactness of the cell walls was retained, even after partial pectin depletion. Whereas in the case of the apple, onion and pumpkin AcUF, these partially pectin-depleted cell walls occurred separately, in the carrot AcUF, they mainly occurred in a clustered manner. The latter observation indicated that the adhesion between the walls by the middle lamella was only slightly impaired by the acid pectin extraction in the case of carrot. Before HPH, the G′ of the suspensions prepared with the AcUFs in deionized water greatly varied among the matrices studied. In these suspensions, the network was likely formed by the interaction between the intact partially pectin-depleted cell walls. The lowest network forming potential was observed for the small particles in the pumpkin AcUF, intermediate for the particles in the carrot and onion AcUF and highest for the particles present in the apple AcUF. The WBC of the AcUFs seemed to be related with the pectin content and retention in the AcUF.

The effect of HPH on the texturizing potential (both the rheological properties and the WBC) was clearly matrix dependent. HPH had a negative effect on the G′ of the apple and carrot AcUF suspensions and no significant effect on the WBC. In the case of the apple AcUF, for which the shear force exerted during HPH at 20 MPa was insufficient to break or disrupt the partially pectin-depleted cell walls, the G′ of the suspension was significantly lower after HPH at 80 MPa than after 20 MPa. For carrot, a substantial decrease in the G′ of the suspension was observed by HPH at 20 MPa, while no significant difference in the G′ was observed between the two intensities used. The possible formation of new interactions and entanglements by the disrupted AcUF of these matrices could thus not result in a network with the same (or higher) stiffness than the network formed by the intact large particles. In contrast, the G′ of the onion and pumpkin AcUF suspensions was unaffected and clearly improved by HPH, respectively, which was associated with a higher WBC of the respective material.

The observed texturizing potential was the result of many (related) microstructural attributes and the chemical composition. As a consequence, one of the most important conclusions of the current study is that the texturizing potential of the partially pectin-depleted fraction of the CWM and the effect of HPH thereon cannot be easily generalized to different fruits and vegetables. Nevertheless, interesting relations and trends were discerned, which allowed to gain more insight into the relation between microstructure, processing by HPH and the resulting functionality as potential texturizing agent.

## Figures and Tables

**Table 1:** Monosaccharide content (mg/g), the degree of methyl esterification (DM) (%), the contribution of the pectin backbone (mol%) and ratio of Glc to UA (−) of the acid-unextractable fractions of the different matrices ± standard deviation (*n* = 2 × 2); <d.l. = below detection limit.

	Apple	Carrot	Onion	Pumpkin
**Monosaccharide composition** **(mg/g)**				
Fuc	7.1 ± 0.9	<d.l.	0.6 ± 0.2	<d.l.
Rha	7.0 ± 0.6	8.4 ± 0.3	3.5 ± 0.1	4.7 ± 0.2
Ara	24.7 ± 0.8	15.9 ± 0.1	4.9 ± 0.3	3.8 ± 0.8
Gal	56.9 ± 3.6	56.6 ± 4.6	77.6 ± 3.9	52.5 ± 4.7
Glc	454.7 ± 14.7	382.8 ± 11.4	425.7 ± 16.6	470.7 ± 19.3
Xyl	70.3 ± 6.5	17.3 ± 0.7	36.8 ± 1.6	35.3 ± 5.9
Man	24.7 ± 1.5	25.8 ± 2.0	17.4 ± 0.8	17.1 ± 3.9
UA	143.2 ± 4.9	193.5 ± 9.0	102.6 ± 4.0	91.4 ± 3.5
**Other compositional and structural properties**				
DM (%)	37.0 ± 2.4	38.4 ± 1.8	40.4 ± 4.3	53.4 ± 3.5
Contribution of the pectin backbone (UA + Rha) (mol%)	17.6 ± 0.7	27.2 ± 1.3	14.8 ± 0.7	13.3 ± 0.6
Ratio of Glc to UA (−)	3.4 ± 0.2	2.1 ± 0.1	4.5 ± 0.2	5.5 ± 0.3

## Data Availability

The data are contained within the article.

## Abbreviations

The following abbreviations and symbols were used in the manuscript.

AcUF	acid-unextractable fraction
AIR	alcohol-insoluble residue
Ara	arabinose
CWM	cell wall material
DM	degree of methyl esterification
Fuc	fucose
Gal	galactose
Glc	glucose
HPH	high-pressure homogenization
PSD	particle size distribution
Man	mannose
Rha	rhamnose
UA	uronic acid
WBC	water binding capacity
Xyl	xylose
d_4,3_	mean volume-based particle size
G′	storage modulus
G″	loss modulus
